# From Crystal Packing to Molecular Recognition: Prediction and Discovery of a Binding Site on the Surface of Polo-Like Kinase 1**

**DOI:** 10.1002/anie.201008019

**Published:** 2011-03-29

**Authors:** Paweł Śledź, Christopher J Stubbs, Steffen Lang, Yong-Qing Yang, Grahame J McKenzie, Ashok R Venkitaraman, Marko Hyvönen, Chris Abell

**Affiliations:** University Chemical Laboratory, University of CambridgeLensfield Road, CB2 1EW, Cambridge (UK) E-mail: ca26@cam.ac.uk; Hutchison/MRC Research CentreHills Road, CB2 0XZ, Cambridge (UK); Department of Biochemistry, University of Cambridge80 Tennis Court Road, CB2 1GA, Cambridge (UK)

**Keywords:** crystallography, kinases, molecular recognition, proteins

Protein–protein interactions are notoriously difficult to target with small molecules as large, discontinuous surfaces are often involved^1^, ^2^ that can adopt different conformations to interact with diverse binding partners.^3^–^5^ In addition, protein surfaces are inherently flexible, exemplified by reports of hits from high-throughput screens that were found to bind in previously unidentified pockets resulting from surface flexibility.^6^ These factors significantly complicate structure-based drug discovery in the context of protein–protein interfaces.^7^ The ability to understand the flexibility of the protein surface and predict its adaptive changes conditioned by molecular recognition of a ligand would open up new avenues for targeting protein–protein interactions. However, despite significant interest there are few systematic methods to accomplish this.^8^, ^9^

Crystal structures provide molecular insight into the basis of protein–protein interactions. In addition to biologically relevant protein–protein interfaces, crystal-packing interactions between neighboring molecules are present in the crystal lattice, often introducing changes to local regions of the protein as compared to the structures in solution.^10^ Although being similar in their physical nature, crystal contacts can be distinguished from genuine biological interactions in the study of molecular recognition.^11^, ^12^ However, despite forming under non-physiological solvent conditions and protein concentrations, they often induce conformational changes on protein surfaces, which may be used to provide direct evidence of surface flexibility and structural motifs inducing these changes.

We examined the crystal-packing interactions formed by the polo-box domain (PBD) of polo-like kinase 1 (Plk1), a validated anti-cancer target,^13^ whose phosphorylation-dependent protein–protein interactions are crucial for successful progression of the cell through mitosis.^14^, ^15^ The PBD has been well characterized crystallographically, both unliganded and in complex with various phosphopeptides: we have obtained ten new crystal forms, which supplement nine already in the public domain.^16^–^19^ Together, these provided extensive data for crystal-packing analysis.

We have observed an unreported binding site in a number of crystal structures of the PBD, which is formed by the rearrangement of surface residues involved in crystal packing. This site is located close to the phosphopeptide-binding groove, making it potentially accessible for phosphorylated proteins binding to the PBD. We sought to use the information derived from the crystal-packing interactions to identify ligands that could span across the phosphopeptide-binding groove into the newly discovered binding site (Figure 1 a).

**Figure 1:**
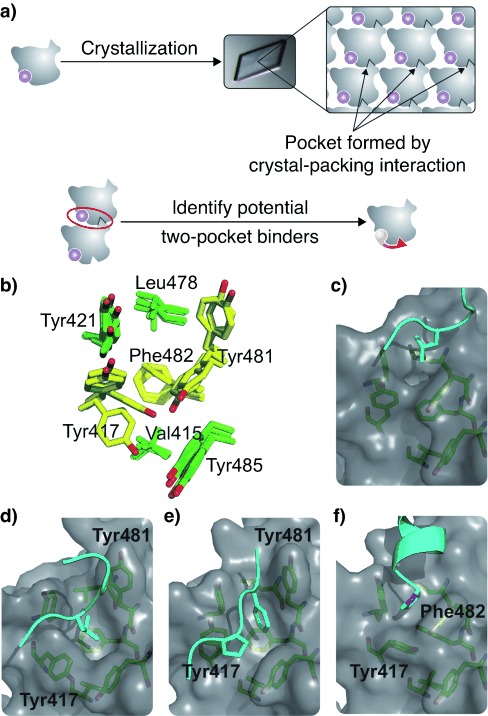
a) Upper: A simplified representation of the packing of PBD (gray)–peptide (purple) complexes in a crystal. Lower: Translation of the crystal-packing interaction into a two-pocket binder. b) Seven residues forming the new site in different conformations observed in the crystal structures; mobile residues shown in yellow. c) The closed hydrophobic pocket from our structure of the unligated PBD. d) The surface of the hydrophobic pocket from our structure of the PBD–MQSpSPL complex. Tyr481_PBD_ and Tyr417_PBD_ change the conformation to accommodate Leu394_SYM_; Leu can also be seen in the pocket in one of the previously published crystal structures.^18^ e) The surface of the hydrophobic pocket from our new structure of the PBD–MQSpTPL complex. Tyr481 is in an open conformation to accommodate Phe8_term_ and Pro6_term_. f) The surface of the hydrophobic pocket from our structure of the PBD–PLHSpTA complex. Tyr417_PBD_ and Phe482_PBD_ change their conformations to accommodate Pro6_term_.

The new binding site consists of seven residues: Val415, Leu478, and Phe482 form the bottom of the pocket whilst Tyr417, Tyr421, Tyr481, and Tyr485 form the sides of the pocket (Figure 1 b). These residues are also conserved in the polo-box domains of the closely related family members Plk2 and Plk3. In most of the published PBD structures the pocket is found in a closed conformation where Tyr481 stacks onto Phe482 and fills the cavity (Figure 1 c; 3P2W). In a number of structures described here, the side-chain conformations of Tyr417, Tyr481, and Phe482 have changed to accommodate hydrophobic residues presented to the pocket by flexible parts of symmetry-related molecules in the crystal (referred to as SYM). In one structure (the PBD complexed with a phosphopeptide, MQSpSPL; 3P35) Tyr481 opens the pocket to accommodate Leu394 from a flexible loop of another PBD protomer. A conformational change in the side chain of Tyr417_PBD_, found at the pocket entrance, closes the pocket around Leu394_SYM_ (Figure 1 d). In another structure (the PBD complexed with the consensus phosphopeptide MQSpTPL; 3P34) Phe8_term_ of the eight N-terminal amino acids of a neighboring PBD protomer is found in the pocket (Figure 1 f). In this case, Tyr417_PBD_ is in an open conformation and the empty space at the edge of the pocket between Tyr417_PBD_ and Tyr485_PBD_ is filled with Pro6_term_ of the Phe8_term_ bearing protomer; this closes the pocket around Phe8_term_. Phe8_term_ occupied a position similar to Leu394_SYM_ in the pocket. A slightly different crystal contact was observed when Pro6_term_ was found in the pocket (Figure 1 f, the PBD complexed with PLHSpTA; 3P2W). In this case Tyr417_PBD_ and Tyr481_PBD_ close over the pocket and the side chain of Phe482_PBD_ rotates by 90°, forming a much shallower cavity.

These observations demonstrate a degree of plasticity within the pocket, which enables it to accommodate different residues. The combination of an adaptable, promiscuous binding pocket with the strong anchoring recognition^20^ for phosphothreonine, suggested that the combination may be important in binding a subset of PBD-interacting proteins, since to date there is little evidence to explain the diverse, yet highly regulated array of PBD-dependent Plk1 activities. To explore this possibility we performed a bioinformatic search of previously identified phosphorylation-dependent PBD-interacting proteins.

We compiled a database of 631 proteins previously shown to interact with the PBD (see Supporting Information).^21^, ^22^ A search string involving the previously reported phosphopeptide groove “consensus” recognition sequence^23^ separated by up to three residues from the putative hydrophobic pocket binder (phenylalanine or leucine) was formulated and applied to the database (Figure 2).

**Figure 2:**
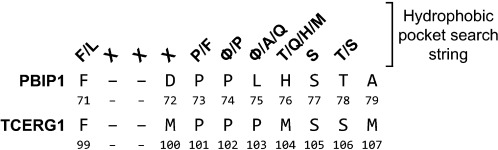
Comparison of the search string used to identify potential hydrophobic pocket-binding proteins and the matched sequences in PBIP1 and TCERG1. X, spacer residues; Φ, hydrophobic residue.

Only 2 out of 631 proteins—TCERG1 (transcription elongation regulator 1) and PBIP1 (polo-box interacting protein 1)—met our search criterion. PBIP1 is involved in the localization of Plk1 to the kinetochores and its phosphorylation-dependent interaction with the PBD involving pThr78_PBIP1_ has been characterized.^24^ Interaction between TCERG1 and PBD has not been characterized in detail before. Interestingly both proteins contain the sequence FXP, resembling the sequence involved in one of the observed crystal contacts (Figure 1 e).

We chose a fluorescence-based thermal stability assay as the first-line screening tool to study the interaction of PBIP1 and TCERG1-derived phosphorylated peptides with the PBD. For a related series of compounds, the thermal shift can be correlated with ligand binding affinity.^25^ To test whether the hydrophobic pocket is involved in peptide binding the assay was performed for both wild-type PBD and a double mutant (Y417A/Y421A), designed to diminish binding to the hydrophobic pocket. Only the PBIP1-derived peptide showed significant difference in thermal shifts for the wild-type (14.4 °C) and mutant protein (10.1 °C), so we decided to concentrate our efforts on this. We further investigated the influence of Phe71_PBIP1_ on its interaction with the PBD (Table 1). The thermal shifts of 9.7 °C observed for 72-DPPLHSpTA-79 (lacking Phe71), and 10.5 °C observed for 71-ADPPLHSpTA-79 were significantly lower than the one observed for 71-FDPPLHSpTA-79 and showed no significant difference between the wild-type and mutant protein.

**Table 1:** A summary of thermal stability and ITC measurements performed for different peptides

Peptide	Thermal shift [°C]			*K*_D_	Δ*H*
	Wild-type	Y417A/Y421A	Δ	[µm]	[kcal mol^−1^]
FMPPPMS**pS**M	2.0	2.0	0.0	515	−18.7
FDPPLHS**pT**A	14.4	10.1	4.3	0.25	−17.6
ADPPLHS**pT**A	10.5	10.7	−0.2	1.32	−15.7
DPPLHS**pT**A	9.7	10.1	−0.4	2.14	−17.1

These results suggest that the interaction between Phe71 and the hydrophobic pocket plays an important role in PBIP1–PBD binding. Our thermal stability studies were further supported by isothermal titration calorimetry (ITC) measurements, which showed an increased affinity for the Phe71-bearing peptide. The role of Phe71 and the hydrophobic pocket in the binding of PBIP1 was confirmed by solving crystal structures of the PBD of human Plk1 in complex with 71-FDPPLHSpTA-79 (3P37) and 72-DPPLHSpTA-79 (3P36).

When DPPLHSpTA is bound to the protein, the hydrophobic pocket adopts a closed conformation and does not participate in peptide binding (Figure 3 a). However, in the structure of the 71-FDPPLHSpTA-79–PBD complex, Phe71_PBIP1_ is found in the hydrophobic pocket (Figure 3 b). The side chain of Tyr481_PBD_ rotates away from the domain to open the pocket, and the space between Tyr417_PBD_ and Tyr485_PBD_ is filled by Pro73_PBIP1_, closing the pocket around the side chain of Phe71_PBIP1_. The positions of Phe71_PBIP1_ and Pro73_PBIP1_ within the pocket are very similar to those observed previously for Phe8_term_ and Pro6_term_ in the crystal contact, despite the reversed direction of the peptide chain (Figure 1 e and 3 c). Linker residues Asp72_PBIP1_ and Glu7_term_ also adopt similar solvent-exposed conformations, but only the side chain of Asp72_PBIP1_ is ordered; the backbone nitrogen atom of this residue forms a hydrogen bond with the hydroxy group of Tyr417_PBD_.

**Figure 3:**
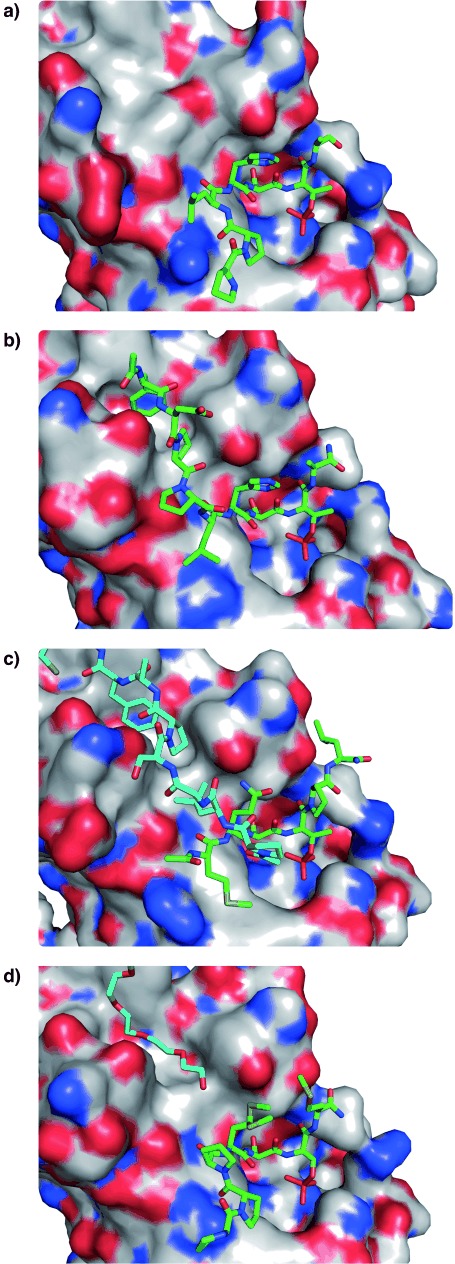
The structures of the PBD in complex with a) DPPLHSpTA; b) FDPPLHSpTA; c) MQSpTPL (the crystal contact with phenylalanine in the pocket was first observed in this structure—see Figure 1 e); d) FMPPPMSpSM peptides. The peptides are shown in green and the surface of the PBD is shown, flexible *N*-terminal tail of symmetry-related protomer in c) and polyethylene glycol in d) are shown in cyan.

These structures provide a rationale for the increased affinity of the longer peptide to the PBD, and show how Phe71_PBIP1_ is inserted in the pocket previously identified by analyzing crystal contacts in the PBD structures. The crystal structure of the TCERG1 derived peptide, 99-FMPPPMSpSM-107 (3Q1I), confirmed the lack of hydrophobic pocket binding and also provided evidence to rationalize this. Pro103_TCERG1_ in TCERG1 occupies the place of Leu75_PBIP1_ in PBIP1 and imposes a conformational restraint that prevents the FMP motif from turning into the hydrophobic pocket, which is occupied by polyethylene glycol used in the crystallization buffer (Figure 3 d).

This analysis of crystal packing interactions has identified a new binding site on the surface of the PBD and novel peptide binding modes. The binding site has been confirmed using biophysical techniques, mutagenesis, and X-ray crystallography, and has been shown to participate in binding to a peptide derived from a biologically relevant ligand, PBIP1. This highlights the need for the ongoing functional in vitro and in vivo studies to elucidate the exact role of the newly discovered hydrophobic pocket in Plk1, as well as in the related family members Plk2 and Plk3. Furthermore, our findings are expected to inform drug discovery efforts targeting the PBD of Plk1.

Given the ever-increasing number of crystal structures in the public domain, we believe that the application of similar analyses of crystal-packing interactions has the potential to provide further valuable insights into molecular recognition at protein–protein interfaces.

## Supplementary material

Detailed facts of importance to specialist readers are published as ”Supporting Information”. Such documents are peer-reviewed, but not copy-edited or typeset. They are made available as submitted by the authors.
