# Supplementary material for: From Crystal Packing to Molecular Recognition: Prediction and Discovery of a Binding Site on the Surface of Polo-Like Kinase 1
Source: Angew Chem Int Ed Engl. 2011 Mar 29;50(17):4003–6. doi: 10.1002/anie.201008019 (PMC3555362; doi:10.1002/anie.201008019)
Supplement: Supplementary file 1 [file anie0050-4003-SD1.pdf]

Angewandte Chemie
Eine Zeitschrift der Gesellschaft Deutscher Chemiker

Supporting Information

© Wiley-VCH 2011

69451 Weinheim, Germany

From Crystal-Packing to Molecular Recognition: Prediction and Discovery of a Binding Site on the Surface of Polo-Like Kinase 1**

*Paweł Śledź, Christopher J. Stubbs, Steffen Lang, Yong-Qing Yang, Grahame J. McKenzie, Ashok R. Venkitaraman, Marko Hyvönen, and Chris Abell**

anie_201008019_sm_miscellaneous_information.pdf

Protein preparation

General considerations

For biophysical measurements full length polo-box domain (residues 345-603) of Plk1 was used. It was cloned into pHAT2 vector to obtain a construct encoding a non-cleavable N-terminal His_6-tag (pHAT2::PBD345-603). For crystallisation purposes, only the ordered part of the PBD was used, hence a construct containing residues 371-594 was designed and cloned into the pGEX-6p-1 vector (pGEX-6p-1::PBD371-594).

His_6-PBD345-603 WT

C41(DE3) *E. coli* cells were transformed with plasmid pHAT2::PBD345-603 were grown to OD_{600} = 0.6 and induced overnight at 20 ^{o}C with 0.2 mM IPTG. Cells were harvested by centrifugation (12,000g, 4°C, 15 min) and subsequently resuspended in 50 mM Tris pH 8.0, 200 mM NaCl, and 0.07% (v/v) BME. A tablet of protease inhibitor cocktail was added and cells were lysed using Emulsiflex C5 homogeniser, and the cell debris pelleted by centrifugation (35,000 g, 4°C, 30 min). The supernatant was loaded onto 5 mL HisTrapFF column (GE Healthcare). The column was washed extensively with lysis buffer, then with lysis buffer supplemented with 100 mM imidazole. Protein was then eluted with 50 mM Tris pH 8.0, 200 mM NaCl, 0.07% (v/v) BME and 250 mM imidazole. Relevant fractions were pooled, concentrated and applied to Superdex 75 16/60 gel filtration column equilibrated with 50 mM Tris pH 8.0, 200 mM NaCl, 1mM EDTA and 1 mM DTT, The final yield was approximately 50 mg of pure protein per litre of *E.coli* culture. Protein was concentrated to 8-10 mg/mL and stored at –80 ^{o}C. Nanospray mass spectrometry analysis revealed that the methionine encoded by the start codon was no longer present in the obtained protein product.

His_6-PBD345-603 Y417A/Y421A mutant

Site-directed mutagenesis of PBD was done with QuikChange site-directed mutagenesis procedure (Stratagene) using WT PBD construct as template. Protein expression and purification was carried out as for the WT protein.

GPLGSPEF-PBD371-594

BL21(DE3) or C41(DE3) *E. coli* cells with transformed plasmid pGEX-6p-1::PBD371-594 were grown to OD_{600} = 0.6 and induced overnight at 20 ^{o}C with 0.1 mM IPTG. Cells were harvested by centrifugation (12,000 g, 4 °C, 15 min) and subsequently resuspended in 50 mM HEPES pH 7.5, 200 mM NaCl, 2 mM EDTA and 1 mM DTT. A tablet of protease inhibitor cocktail was added and cells were lysed using Emulsiflex, and the cell debris pelleted by centrifugation (35,000 g, 4°C, 30 min). The supernatant was applied to 5 mL of glutathione Sepharose resin (GE Healthcare). The resin was washed with 100 mL of the lysis buffer, then 50 mL of 10 mM HEPES pH 7.5, 500 mM NaCl, 2 mM EDTA and 1 mM DTT. The GST-tag was cleaved using 1:50 (v/v) of rhinovirus 3C protease (1 mg/mL) (2 h, 4 °C). The cleavage product was subjected to gel filtration using Superdex 75 16/60 column equilibrated with 10 mM HEPES pH 7.5, 500 mM NaCl, 1 mM EDTA and 1 mM DTT, concentrated to 10-15 mg/mL and stored at -80 ^{o}C.

Phosphorylated peptides

Sources of peptides are summarized in Table S1. Peptides **1 – 3** were synthesised in-house on a Liberty Microwave Peptide Synthesiser (CEM Corporation, Mathews, NC), as previously reported.[1] Crude peptides were purified by reverse-phase HPLC on a Gilson GX-271 equipped with a Gilson 171 diode array detector using a Polaris C8-A (5 µm; 4.6 x 300 mm (analytical), 21.2 x 300 mm (preparative); Varian, inc.) column at 1 mL/min (analytical) or 21 mL/min (preparative) using a linear gradient of 5 % to 30 % B over 30 min. The solvent system used was A (0.1 % (v/v) TFA in H_2O) and B (0.1 % (v/v) TFA in acetonitrile). Peptide identities were confirmed by MALDI-TOF-MS (ABI 4700 Proteomics Analyzer, Applied Biosystems) and amino acid analysis.

Peptides **4 - 7** were synthesised externally and used without further purification.

Table S1. Source of peptides used in this study

	Peptide sequence	Source
1	Ac-PLHSpTA-NH_2	Synthesised in house
2	Ac-DPPLHSpTA-NH_2	
3	Ac-ADPPLHSpTA-NH_2	
4	Ac-FDPPLHSpTA-NH_2	PNAC facility, Department of Biochemistry, University of Cambridge
5	Ac-MQSpTPL-NH_2	Designer Bioscience
6	Ac-MQSpSPL-NH_2	
7	Ac-FMPPPMSpSM-NH_2	

Thermal shift measurements

Thermal shift experiments were performed on a Roche Light Cycler 480 using 96-well plates. The samples were heated in the thermal cycler from 37 to 62 °C with a heating rate of 0.3 °C/min. The thermal unfolding event was observed by adding 2.5x Sypro Orange dye, which was excited at 490 nm and emission was detected at 530 nm. (Invitrogen) Standard experiments were conducted using a total of 100 µL solution per well, consisting of 90 µL buffer, 10 µL 1 mM peptide solution and 0.05 µL 5000x Sypro Orange solution. In order to determine the optimal buffer solution, several buffers were tested. We focused on obtaining a maximal value for the difference in thermal melting between the free and liganded forms of the protein. Figure S1 show results of these optimisation studies. The optimal conditions were found when using a buffer containing 50 mM MES (2-(*N*-morpholino)ethanesulfonic acid) pH 6.8, 50 mM NaCl and 2 mM EDTA. Figure S2 shows the thermal unfolding traces for PBIP1-derived peptides used to generate the data in Figure 2b.

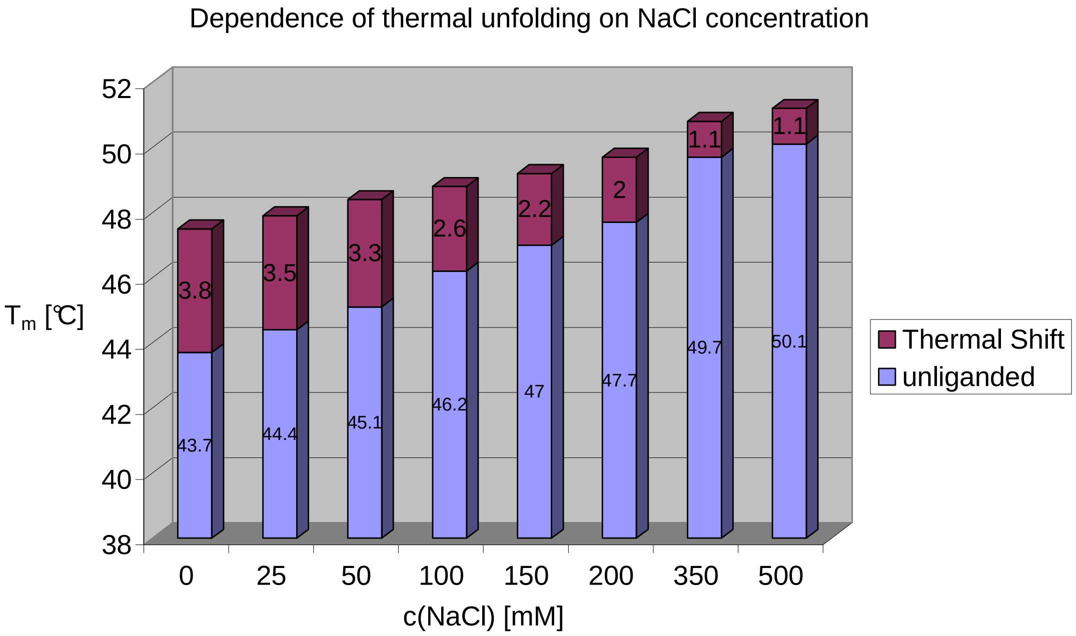

Figure S1. Optimization of the NaCl concentration for the thermal shift experiments.

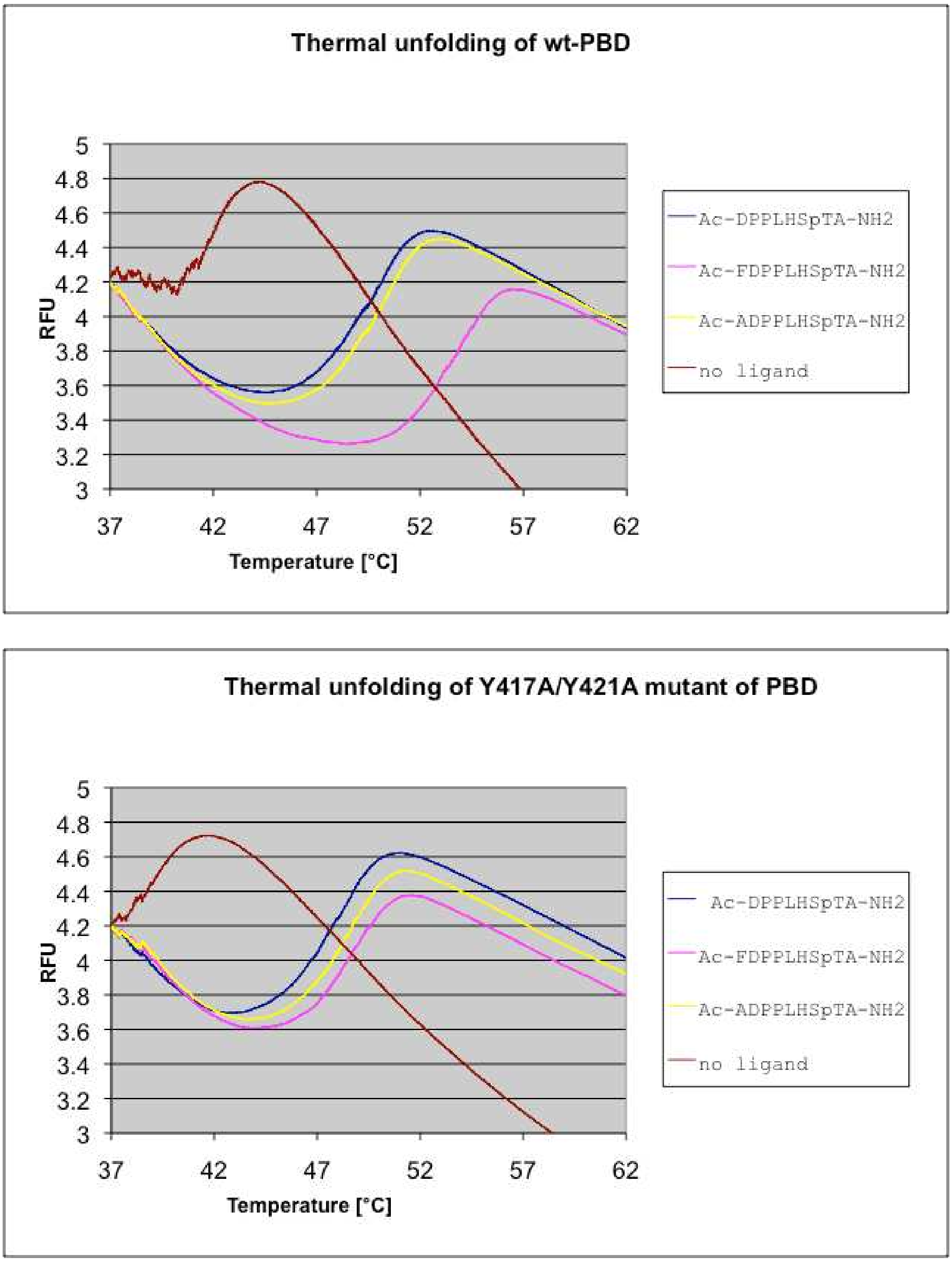

Figure S2. Comparison of thermal unfolding traces for the wild-type and mutated PBD.

Isothermal titration calorimetry

ITC experiments were performed using the iTC200 instrument (Microcal Inc. – GE Healthcare) at 25 °C. His_6-PBD345-603 was loaded into the ITC cell at concentration of 30 μM. Ligands were dissolved in the same buffer to the concentration of 500 μM (except for Ac-FMPPPMSpSM-NH_2, which was used at 2 mM). Typically, 25 injections of 1.5 μL in volume were done over a period of 30 min. Data was fitted to single binding site model using the Origin software package provided by the manufacturer. ITC traces together with their interpretation are presented below.

Ac-ADPPLHS<u>pT</u>A-NH_2 | Ac-DPPLHSpTA-NH_2

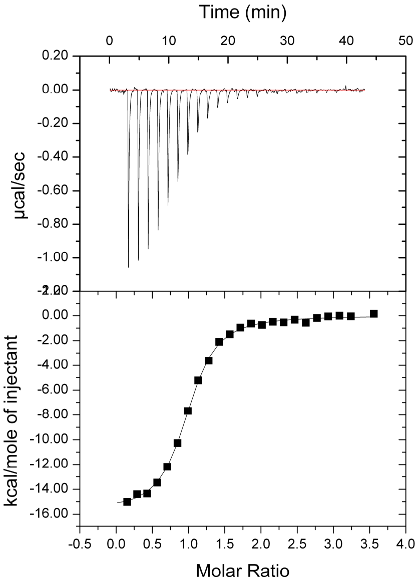

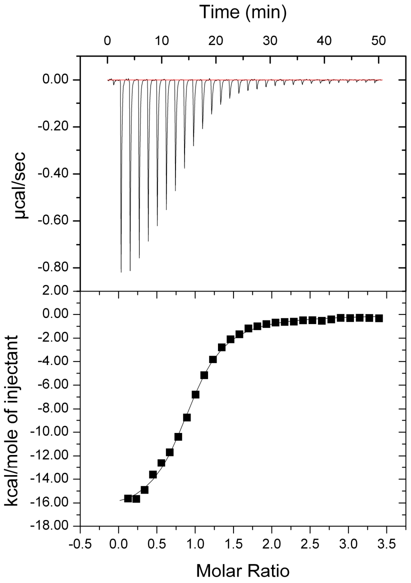

	Ac-ADPPLHSpTA-NH_2	Ac-DPPLHSpTA-NH_2
K_D	1.3 ± 0.2 µM	2.2 ± 0.4 µM
ΔH	–15.8 ± 0.1 kcal/mol	–17.1 ± 0.2 kcal/mol

Ac-FDPPLHSpTA-NH_2 | Ac-FMPPPMSpSM-NH_2

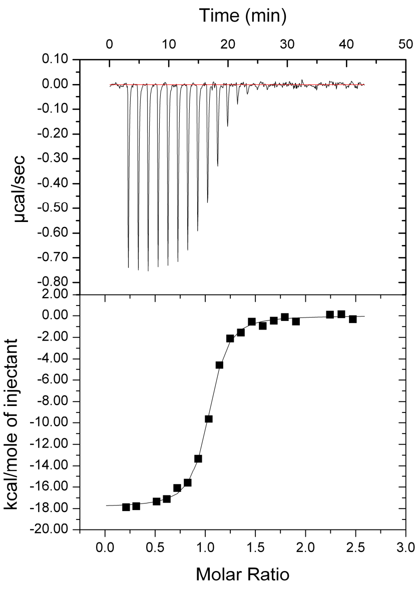

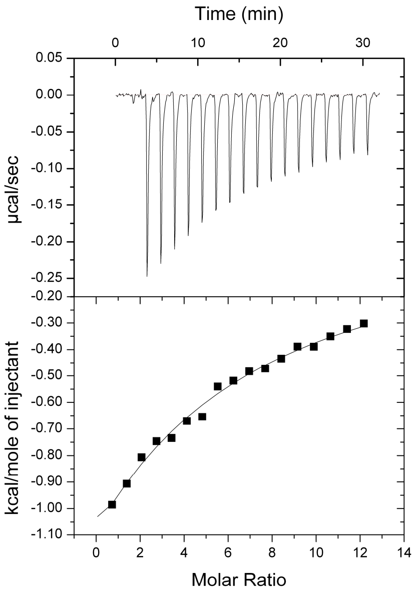

	Ac-FDPPLHSpTA-NH_2	Ac-FMPPPMSpSM-NH_2
K_D	0.25 ± 0.03 µM	520 ± 20 µM
ΔH	–17.8 ± 0.2 kcal/mol	-18.8 ± 0.5 kcal/mol

Bioinformatics analysis

A database of phosphorylation-dependent PBD-interacting proteins was constructed using the data from a mass spectrometric study of the PBD interactome by Lowery *et al.*.[2] These data were supplemented by other known phosphorylation-dependent PBD-interacting proteins (Bub1; BubR1; Cdc25C; FoxM1; HBO1; HsCYK-4; MKLP2; Nedd1-β; PBIP1; PICH; PRC1; TRF1; Vimentin; Wee1A).

All bioinformatic procedures were accomplished using programs in the BLAST suite (NCBI).[3] The amino acid sequences of all 631 proteins were downloaded in FASTA format from the NCBI database and formatted into a BLAST-compatible database using `formatdb`. A search string was then generated for the `seedtop` program, which searches a given protein sequence for the presence of a defined sequence. The program `fastacmd` was used to extract the matching sequences from the hits identified by `seedtop`.

Protein crystallography

Crystallization

Protein was crystallised in either a hanging drop or sitting drop setup by mixing a solution of GPLGSPEF-PBD371-594 or its complex concentrated to 10-15 mg/mL with the well solution in a 1:1 (v/v) ratio. Typically, crystals were obtained within 24–48 hours, except for complex **4** for which crystals appeared after 5 days and complex **3,** which took 14 days to crystallise. Table S2 summarises the conditions for crystallisation experiments.

Table S2. Summary of crystallisation conditions

Complex	Ligand	Conditions
1	—	100 mM HEPES pH 7.5, 1.0-2.0 M $(NH_4)_2SO_4$
2	Ac-MQSpTPL-NH_2	100 mM HEPES pH 7.5, 1.0-2.0 M $(NH_4)_2SO_4$
3	Ac-MQSpSPL-NH_2	0.2 M potassium formate, 20% PEG 3350
4	Ac-PLHSpTA-NH_2	0.2 M K/Na tartrate, 20% PEG 3350
5	Ac-FDPPLHSpTA-NH_2	100 mM MES pH 6.5, 30% PEG 400
6	Ac-DPPLHSpTA-NH_2	100 mM Na/K phosphate pH 6.2, 0.2 M NaCl, 10% PEG 8000
7	Ac-FMPPPLSpSM-NH_2	100 mM MES pH 6.5, 30% PEG 400

Structure solution and refinement

Experimental diffraction data were collected at Swiss Light Source, Diamond Light Source, European Synchrotron Radiation Facility and Soleil Synchrotron. Data were processed with XDS[4] and structures were solved by molecular replacement using Molrep[5] or Phaser[6] from the CCP4 program suite. Models were manually rebuilt with Coot and structures were refined using Refmac.[7] The summary of data collection and refinement is shown in Table S3.

Table S3. Summary of data collection and refinement

**Ligand:**	Ac-DPPLHSpTA-NH_2	Ac-PLHSpTA-NH_2	Ac-MQSpSPL-NH_2	Ac-FDPPLHSpTA-NH_2	Unliganded form	Ac-MQSpTPL-NH_2	Ac-FMPPPMSpSM-NH_2
**PDB Id**	3P36	3P2Z	3P35	3P37	3P2W	3P34	3Q1I
**Data collection**							
X-ray source	ESRF, ID14-1	ESRF, ID14-1	ESRF, ID14-1	ESRF, ID14-1	SLS, PXIII	Diamond, IO3	Soleil, Proxima 1
Wavelength (Å)	0.9334	0.9334	0.9334	0.9334	0.9794	0.9537	0.9184
Space group	$P2_1$	$P2_1$	$P2_1$	$P2_1$	$P2_1$	$P2_12_12_1$	$P2_1$
Cell dimensions							
a, b, c (Å)	35.93 50.12 58.73	34.41 66.83 43.43	58.89 95.83 65.96	58.96 88.66 67.59	33.46 98.88 36.14	33.46 98.88 36.14	35.50 55.50 57.6
α, β, γ (°)	90.0 99.3 90.0	90.0 94.1 90.0	90.0 116.3 90.0	90.0 113.5 90.0	90.0 98.5 90.0	90.0 90.0 90.0	90.0 101.0 90.0
Resolution	57.93 – 1.59 Å	43.31 – 1.79 Å	59.13 – 2.09 Å	62.02 – 2.38 Å	49.45 – 1.66 Å	58.38– 1.40 Å	56.54 – 1.40 Å
(high res shell)	(1.69 – 1.59)	(1.90 – 1.79)	(2.22 – 2.09)	(2.53 – 2.38)	(1.76-1.66)	(1.44-1.40)	(1.44-1.40)
R_{sym}	4.1 (59.7)	6.8 (62.0)	13.9 (61.1)	11.4 (70.2)	5.2 (48.0)	6.9 (68.1)	5.4 (24.7)
I/σI	22.35 (2.31)	16.03 (2.10)	9.69 (2.27)	11.12 (2.15)	16.84 (2.98)	13.36 (1.95)	16.57 (5.79)
Completeness (%)	99.4 (98.4)	99.6 (98.6)	98.1 (96.8)	99.4 (98.5)	96.0 (91.7)	99.0 (99.7)	96.7 (95.1)
Redundancy	3.69 (3.64)	3.74 (3.70)	3.81 (3.76)	3.79 (3.77)	3.80 (3.61)	6.61 (4.48)	4.09 (3.74)
No. reflections	26 218	17 491	36 378	25 445	26 961	50 685	38 959
**Refinement**							
R_{work}/R_{free}	20.2/25.6	21.2/26.4	20.3/27.3	21.9/27.8	20.5/23.2	21.2/22.9	21.4/22.4
PBD molecules in the asymmetric unit	1	1	3	3	1	1	1
No. atoms							
Protein*	1867	1760	5540	5456	1829	1907	1811
Ligand/ion	11	6	30	30	28	16	41
Water	189	146	486	114	201	270	192
RMS deviations							
Bond lengths (Å)	0.024	0.006	0.019	0.007	0.006	0.006	0.006
Bond angles (°)	2.2	0.9	1.9	1.1	1.0	1.0	1.0
Model quality							
B-factors							
Protein	21.0	10.6	10.4	7.8	8.7	13.1	13.3
Ligand/ion	45.9	38.2	40.0	46.1	40.9	38.6	62.7
Water	33.1	20.9	17.3	8.0	21.6	24.6	27.8
Ramachandran							
Favoured	98.1	99.0	97.4	96.9	96.7	97.4	96.7
Allowed	1.9	1.0	2.6	3.1	2.3	2.6	3.3
Outlier	0.0	0.0	0.0	0.0	0.0	0.0	0.0

* Phosphopeptides were treated as protein

References

1. Harris, P.W.R., Williams, G.M., Shepherd, P., Brimble, M.A. *Int. J. Pept. Res. Ther.* **14**, 387 (2008).

2. Lowery, D.M. *et al. EMBO J* **26**, 2262 (2007).

3. Altschul, S.F., Thomas L. Madden, Alejandro A. Schaffer, Jinghui Zhang, Zheng Zhang, Webb Miller, and David J. Lipman *Nucleic Acids Res.* **25**, 3389 (1997).

4. Kabsch, W. *Acta Crystallogr. D. Biol. Crystallogr.* **66**, 125 (2010).

5. Vagin, A., Teplyakov, A. *Acta Crystallogr. D. Biol. Crystallogr.* **66**, 22 (2010).

6. McCoy, A.J. *Acta Crystallogr. D. Biol. Crystallogr.* **63**, 32 (2007).

7. Murshudov, G.N., Vagin, A.A., Dodson, E.J. *Acta Crystallogr. D. Biol. Crystallogr.* **53**, 240 (1997).
